# Magnetic Phase Coexistence and Hard–Soft Exchange Coupling in FePt Nanocomposite Magnets

**DOI:** 10.3390/nano10081618

**Published:** 2020-08-18

**Authors:** O. Crisan, I. Dan, P. Palade, A. D. Crisan, A. Leca, A. Pantelica

**Affiliations:** 1 National Institute for Materials Physics, 077125 Magurele, Romania; palade@infim.ro (P.P.); alina.crisan@infim.ro (A.D.C.); aurel.leca@infim.ro (A.L.); 2 R&D Consulting and Services S.R.L., 023761 Bucharest, Romania; ioan_dan@rd-consultanta.ro; 3 Horia Hulubei National Institute for Physics and Nuclear Engineering, P.O. Box MG-6, 077125 Magurele, Romania; apantel@ifin.nipne.ro

**Keywords:** L1_0_ FePt, nanocomposite magnets, mechanical alloying, exchange coupling

## Abstract

With the aim of demonstrating phase coexistence of two magnetic phases in an intermediate annealing regime and obtaining highly coercive FePt nanocomposite magnets, two alloys of slightly off-equiatomic composition of a binary Fe-Pt system were prepared by dynamic rotation switching and ball milling. The alloys, with a composition Fe_53_Pt_47_ and Fe_55_Pt_45_, were subsequently annealed at 400 °C and 550 °C and structurally and magnetically characterized by means of X-ray diffraction, ^57^Fe Mössbauer spectrometry and Superconducting Quantum Interference Device (SQUID) magnetometry measurements. Gradual disorder–order phase transformation and temperature-dependent evolution of the phase structure were monitored using X-ray diffraction of synchrotron radiation. It was shown that for annealing temperatures as low as 400 °C, a predominant, highly ordered L1_0_ phase is formed in both alloys, coexisting with a cubic L1_2_ soft magnetic FePt phase. The coexistence of the two phases is evidenced through all the investigating techniques that we employed. SQUID magnetometry hysteresis loops of samples annealed at 400 °C exhibit inflection points that witness the coexistence of the soft and hard magnetic phases and high values of coercivity and remanence are obtained. For the samples annealed at 500 °C, the hysteresis loops are continuous, without inflection points, witnessing complete exchange coupling of the hard and soft magnetic phases and further enhancement of the coercive field. Maximum energy products comparable with values of current permanent magnets are found for both samples for annealing temperatures as low as 500 °C. These findings demonstrate an interesting method to obtain rare earth-free permanent nanocomposite magnets with hard–soft exchange-coupled magnetic phases.

## 1. Introduction

During the last decade, a lot of research effort has been dedicated to the quest for new classes of permanent magnets, RE-free and capable of operating at high temperatures. Among the possible solutions, FePt stands as one of the most promising due to significant magnetocrystalline anisotropy, high coercivity and remanence of its ordered tetragonal L1_0_ phase. In its nanoparticulate form, FePt has good catalytic properties, high corrosion resistance and is biocompatible. For these reasons, FePt nanoparticles have been intensively investigated for uses in biomedicine (magnetic hyperthermia [1], MRI imaging [2] and targeted drug delivery [3]) and in catalysis [4] as sensors [5] or biosensors [6]. Various chemical and physical synthesis methods, such as thermal decomposition using high-temperature boiling point solvents [7], polyol [8], microemulsions [9], microwave processing [10], sonochemistry [11], pulsed plasma in liquid [12], or inert gas condensation [13], have been used to obtain FePt nanoparticles with well-defined shape and controllable sizes. Various other reports deal with the specific link between synthesis methods and magnetic performances in a large range of magnetic materials [14,15,16,17,18,19,20,21,22,23,24,25,26]. As a building block for various devices, FePt is also of interest. Self-assembled nanostructures realized using FePt nanoparticles onto block copolymers templates [27] are considered for high density magnetic recording in the near future using either the heat-assisted magnetic recording method (HAMR) [28] or as self-organized magnetic array (SOMA) bit-patterned media [29]. On the other hand, as bulk materials, FePt-based alloys are very promising as permanent magnets operating in extreme conditions when taking into account some of their attributes, such as corrosion resistance at high temperatures, increased Curie temperature when with rare earth (RE)-containing permanent magnets (477 °C), high magnetocrystalline anisotropy (10^7^ MJ/m^3^) and saturation magnetization (1.4 T) and considerable energy product. Of importance for a series of applications, FePt-based alloys also show increased hardness and Young modulus [30]. As chemical routes or vacuum deposition methods do not allow production of a high amount of material needed for certain applications, other techniques can be envisaged. Non-equilibrium metallurgical methods such as melt-spinning [31] or ball-milling [32] are proven to be prolific in obtaining sufficiently high amounts of homogeneous Fe-Pt alloys. Furthermore, there is much more versatility in metallurgical methods since they allow for slight changes in composition and doping with a small amount of other elements in order to promote the formation of the L1_0_ phase directly from the synthesis without the need for additional annealing, or to promote the formation of additional magnetic phases, needed for instance to construct a nanocomposite exchange-spring FePt-based magnet. Mechanical alloying via ball milling is one of these non-equilibrium techniques which allows, for instance, one to obtain industrially significant quantities of nanocomposite powders in a single run. Moreover, further processing of the FePt-based nanocomposites into geometries and shapes (discs; cylinders) required for applications can be conducted by using, e.g., spark plasma sintering [32,33]. Several studies on FePt nanocomposites made by ball milling have been reported. Surfactant-assisted ball milling of Fe-Pt using oleylamine and oleic acid was employed in [34], however, it proved to be somewhat ineffective in obtaining L1_0_ FePt with high coercivity. Lyubina et al. [35] reported synthesis by ball milling of Fe and Pt powders at liquid nitrogen temperature in order to counteract Pt tendency to stick onto balls during milling. However, after annealing, they obtained a mixture of hard magnetic L1_0_ and soft magnetic cubic FePt phases. Hu et al. [36] obtained L1_0_ FePt from ball milling of a molecular complex precursor and the method was deemed suitable for diminishing the formation temperature of the L1_0_ phase down to 450 °C. In the present work, we report on the coexistence of hard L1_0_ and soft L1_2_ FePt phases, we follow the evolution with the temperature of the structural phase transition with occurrence of the L1_0_ phase at early stages of annealing by means of temperature-dependent X-ray diffraction of synchrotron radiation, and we show that the coexistence of the two phases, partially exchange-coupled at 400 °C, leads to a fully coupled exchange spring magnet at temperatures as low as 500 °C. 

## 2. Experiment

Two FePt compounds with compositions close to the equiatomic ratio, Fe_53_Pt_47_ and Fe_55_Pt_45_, were synthesized using ball milling, starting from constituent powders Pt (99.9+%, 325 mesh, Alfa Aesar, Thermo Fisher GmbH, Kandel, Germany) and Fe (99.9%, iron powder spherical < 10 µm, Alfa Aesar). A Retsch PM400 (Retsch GmbH, Haan, Germany) planetary mill dotted with stainless steel vials of 125 mL was used for the synthesis process. Each mixture of constituent Fe and Pt powders weighed about 3.3 g. During milling, one 20 mm stainless steel ball and eight 10 mm stainless steel balls were loaded in the vials together with the composite powder, ensuring a ball-to-powder mass ratio of 20:1. Milling cycles, 10 min long, were separated by a 3 min stop to avoid the powder overheating and element segregation. The direction of rotation was reversed at each pause. A small amount of hexane (30 mL for each vial) was used to cover the powder and balls in the vials with the aim of diminishing the Pt adherence to the balls and loss of effective powder mass of the final composition. The total effective milling time was 8 h at a rotation frequency of 350 rpm. The powders were loaded to—and afterwards extracted from—vials under a protective argon atmosphere (<1 ppm O_2_, <1 ppm H_2_O), and operations were performed in an MBraun Labstar (MBraun Inertgas-Systeme GmbH, Garching, Germany) glove box. With the purpose of following the disorder–order transformation that conveys the formation of the hard magnetic tetragonal L1_0_ phase, the as-milled powders were subsequently annealed under argon flow (99.9999% purity, 100 mL/ min) for 1.5 h at 400 °C and 500 °C for samples Fe_53_Pt_47_ and Fe_55_Pt_45_, respectively. The composition of the milled alloys was verified using proton induced X-ray emission spectrometry (PIXE). This analysis was carried out using a 3 MeV proton beam generated at the 3 MV Tandetron accelerator of National Institute for Physics and Nuclear Engneering, NIPNE. The crystalline structure of the composite powders was investigated by powder X-ray diffraction (XRD) using a Bruker D8 Advance (Bruker Corporation, Billerica, MA, USA) diffractometer with Cu K*_α_* radiation in *θ*–2*θ* geometry. The evolution with the temperature of the disorder–order phase transformation was monitored with temperature-dependent X-ray diffraction of synchrotron radiation. The experiments were performed between 200 °C and 600 °C using monochromatic radiation (*λ* = 0.066 nm) at the X04 SA materials science beamline at the Swiss Light Source (Paul Scherrer Institute, Villigen, Switzerland). The heating during measurements was carefully performed and, upon reaching the set temperature value, an additional 2 s settle time was allowed for temperature stabilization at the desired value within 1 °C. The measurement protocol involves the collection of two diffractograms for 4 s exposure at two different detector positions followed by merging the two results into one pattern to remove the geometrical effects. The total exposure time of the sample at a given temperature was about 10 s. Magnetometry measurements were performed in order to characterize the magnetic properties of the samples. Hysteresis loops at 27 °C were acquired with a Quantum Design (Quantum Design Europe GmbH, Darmstadt, Germany) SQUID magnetometer running under RSO (reciprocate sample option) mode. The spin structure was investigated using ^57^Fe Mössbauer spectroscopy performed at room temperature with a linear spectrometer in a conventional set-up in transmission geometry. The gamma source we used was a ^57^Co source in the Rh matrix.

## 3. Results and Discussion

The chemical composition of the resulted powder was verified using proton induced X-ray emission spectroscopy (PIXE). Proton-induced X-ray emission is an elemental analysis technique which uses a highly energetic beam of heavy charged particles (usually protons of kinetic energy between 1 and 4 MeV) to produce an element-specific X-ray emission from solid samples. Compared to other compositional analysis techniques, for instance, energy-dispersive X-ray spectroscopy, PIXE has the advantage of low detection limits of 0.1–10 mg/kg (or 10^−5^–10^−7^) of the concentration, especially for low Z elements. The composition of the as-milled alloys was found to be very close to nominal composition, within 0.5 at%. The alloys compositions that resulted from PIXE are Fe_52.6_Pt_47.4_ and Fe_55.3_Pt_44.7_. No oxygen or carbon contamination was found in the as-milled alloys, as proven by structural analysis. 

**Figure 1:**
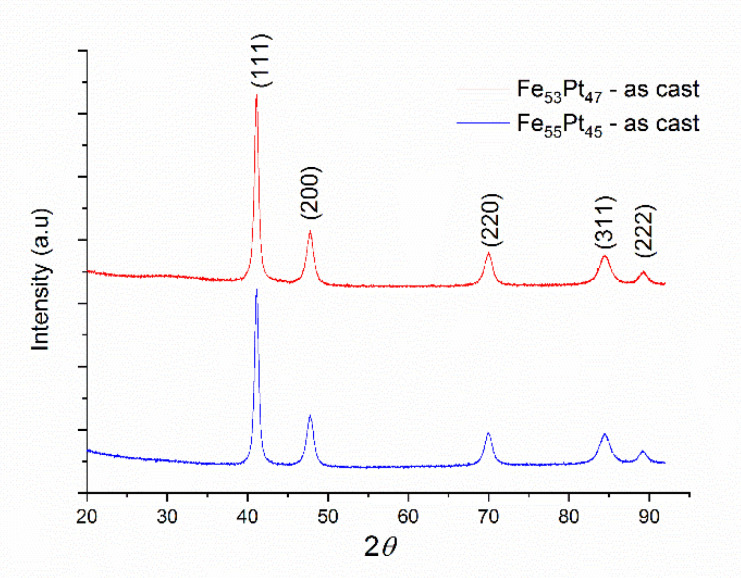
XRD diffractograms for Fe_53_Pt_47_ and Fe_55_Pt_45_ as-milled powders.

Both as-milled samples Fe_53_Pt_47_ and Fe_55_Pt_45_ were structurally investigated by XRD, and the obtained diffractograms are shown in Figure 1. It can be seen that the diffractograms for both samples are quite similar, as expected, and present several Bragg lines, typical for nanocrystalline materials. Full-profile Rietveld-type analysis was performed and we were able to index these peaks as belonging to the (111), (200), (220), (311) and (222) reflections of the face-centered-cubic A1 FePt phase. 

The quantitative analysis was conducted by full profile refinement of the patterns using a Rietveld-type fitting software named MAUD (Materials Analysis Using Diffraction) [37]. The fit provided the accurate peak positions and full width at half maximum (FWHM) of the peaks. From these values, we calculated the average grain size and the lattice parameters. The average grain size for the Fe_53_Pt_47_ was calculated to be 22 nm, while for Fe_55_Pt_45_ the calculated value was quite close: 27 nm. The calculated lattice parameters were also very close to each other: 3.803 Å for Fe_53_Pt_47_ and 3.804 Å for Fe_55_Pt_45_.

**Figure 2:**
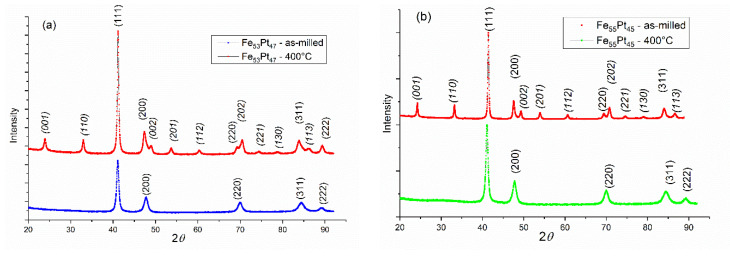
XRD diffractograms for as cast samples of (**a**) Fe_53_Pt_47_ and (**b**) Fe_55_Pt_45_ powders annealed at 400 °C.

In Figure 2a,b we comparatively plotted the X-ray diffractograms of the as-milled samples and annealed at 400 °C both the Fe_53_Pt_47_ and Fe_55_Pt_45_ samples. It can be observed that many more Bragg lines appear after annealing. The MAUD analysis and peak indexation allowed us to unambiguously assign these supplementary peaks to the face-centered tetragonal L1_0_ FePt phase. Most significant are the two (001) and (110) so-called main superlattice peaks whose occurrence is a widely accepted criterion of correct identification of the tetragonal phase. However, the occurrence of the L1_0_ phase is proven also by some other superlattice peaks and the tetragonal splitting of the convoluted Bragg peaks. In the cubic A1 lattice, the (200), (220) and (311) Bragg lines are the second most intense peaks after the main (111) peak. However, due to tetragonal distortion, after the disorder–order transition and occurrence of the L1_0_ phase, these Bragg lines split as follows: (200) into (200) and (002); (220) into (220) and (202); and (311) into (311) and (113). The more obvious the splitting, the more significant the phase transformation and the more abundant the formed L1_0_ phase. For better comparison, all the Bragg lines that are unambiguously attributed to the tetragonal L1_0_ phase are denoted in italics. 

**Figure 3:**
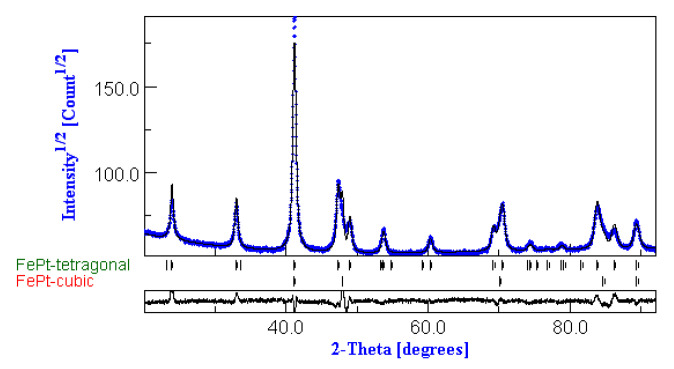
MAUD full-profile fitting of the diffractogram of sample Fe_53_Pt_47_ annealed at 400 °C.

Quantitative full-profile analysis was performed on the annealed samples, with the aim of quantizing the amount of the L1_0_ phase formation in the two investigated samples. The fitting results are shown in Figure 3 for the Fe_53_Pt_47_sample annealed at 400 °C, where both experimental (black dots) and the fitting (blue line) are plotted. The fitting model included two crystal structures: the face-centered-cubic *fcc* FePt and the tetragonal L1_0_ FePt. The fitting shows good agreement with the experiment, and all major Bragg lines are accounted for. The results of the annealed samples suggest that the powders consist of a nanoscale size-mixed structure of *fcc* and L1_0_ FePt phases with probably well-formed L1_0_ grains separated by less ordered regions of cubic symmetry. Similar results were also obtained for the Fe_55_Pt_45_sample annealed at 400 °C. The fitting quantitative results are given in Table 1. 

As can be seen, the lattice parameter does not change a lot with the small changes in composition in the as-milled samples, and the average grain size, calculated using the integral breadth method, is between 20–30 nm for both samples. The annealed samples, which consist of both cubic and tetragonal phases, show a slightly lower lattice parameter for the cubic phase than the as-milled case, while the tetragonal phase lattice parameters (*a* and *c*) are very similar for the two annealed samples. This indicates that the microstructure of the two samples is very much alike, with very little influence of the compositional changes that differentiate the two as-milled powders. The L1_0_ phase is highly predominant in both annealed samples (between 72% and 78%).

**Figure 4:**
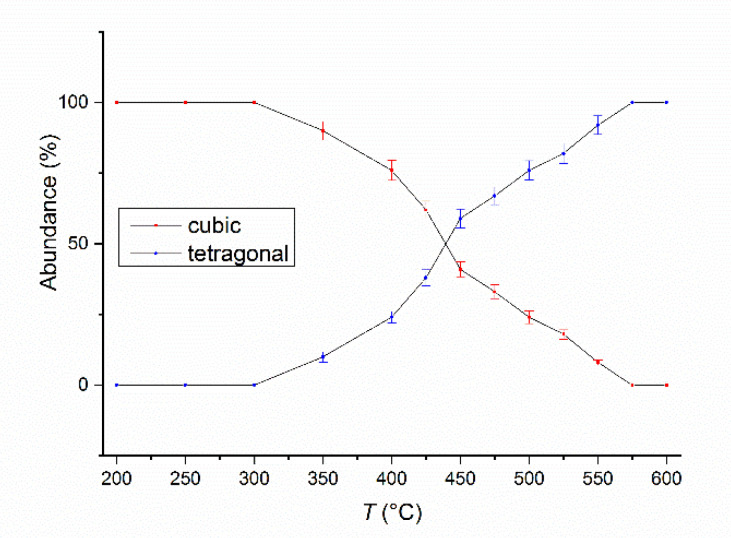
The relative ratio of the cubic and tetragonal phase abundance as a function of temperature, as obtained from the full-profile fitting of the synchrotron X-ray diffractograms.

**Figure 5:**
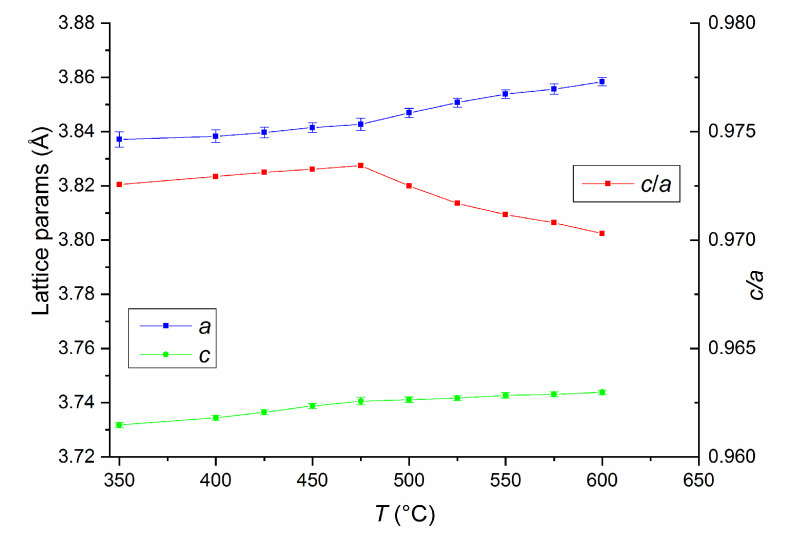
Tetragonal lattice parameters (*a* and *c*) and the *c/a* parameter as a function of temperature obtained from the full-profile fitting of the synchrotron X-ray diffractograms.

In order to monitor the phase structure and the evolution of the disorder–order phase transformation, a temperature-dependent synchrotron X-ray diffraction study was undertaken. X-ray diffractograms on the as-milled Fe_55_Pt_45_ sample were recorded after heating the sample at temperatures ranging from 200 °C to 400 °C in 50°C steps and from 400 °C to 600 °C in 25 °C steps. For the first three recorded diffractograms, at 200 °C, 250 °C and 300 °C, only the peaks of the cubic FePt phase are observed. Starting with 350 °C, the superlattice peaks of the L1_0_ phase start to appear. For this temperature, the L1_0_ phase abundance was estimated from full-profile fitting to be about 10%. Upon heating at 400 °C, this value increases even more, reaching about 24%. Starting with 450 °C, the L1_0_ phase becomes predominant, its abundance being larger than that of the parent cubic phase (59:41). The phase transformation towards complete formation of the L1_0_ phase is steady as we increase the temperature of the XRD study, and this transformation is complete at about 575 °C. The relative abundance of the cubic and tetragonal phases in the as-milled Fe_55_Pt_45_ sample vs. the temperature of the XRD experiment is presented in the Figure 4. The tetragonal L1_0_ structure remains single-phased in the alloy and is thermally stable up to 600 °C. Of particular interest for the stability of the structure and phase evolution with temperature is also the monitoring of the lattice parameters of the tetragonal phase as a function of temperature. The evolution of the tetragonal lattice parameters (*a* and *c*) as revealed from the fitting of XRD data with the temperature of up to 600 °C is shown in Figure 5. Both lattice parameters, *a* and *c,* increase slowly at the same pace between 350 °C and 475 °C. From 475 °C to 600 °C, the temperature at which the L1_0_ phase becomes predominant in the sample, *a* parameter start to increase more abruptly while the increase in *c* parameter become slower. As a consequence, the tetragonality ratio *c/a* exhibits an abrupt decrease from 475°C. This measurement indicates that there is a predominant in-plane expansion of the tetragonal lattice from 475 °C. One can observe that, between 475 °C and 600 °C, the total in-plane expansion along the *a* = *b* axes (Δ*a* ≈ 0.015 Å) is 5 times larger than the total expansion along the *c* axis (Δ*c* ≈ 0.003 Å). As a consequence, we note that the thermal expansion of the tetragonally distorted unit cell is strongly anisotropic, occurring essentially in-plane. This phenomenon has also been observed in ternary L1_0_ phases in Fe-Mn-Pt [38] and is in agreement with thermal expansion behavior of Fe-Pt-Ag-B alloys [39]. The corresponding tetragonality ratio (*c*/*a*) is shown also in Figure 5. It can be seen that there is an optimal tetragonality ratio at about 475 °C where the value reaches a maximum of about 0.9735. Beyond this temperature, the tetragonality ratio decreases to about 0.97 at 600 °C, due to the in-plane thermal expansion of the unit cell. However, the variation in the tetragonality parameter only reaches 3.5% over the whole temperature range, and a value of 0.97 tetragonality ratio stands for a high degree of tetragonal ordering of the L1_0_ phases with important consequences on the overall magnetic behavior of the alloy. The *c*/*a* ratio defines the degree of tetragonal distortion of the atomic structure, thus, it is a valuable parameter of interest, as it is correlated to the degree of magnetocrystalline anisotropy (MCA) of the L1_0_ phase [40].

Another parameter of interest for defining the high degree of ordering of the tetragonal phase is represented by the crystallographically coherent (or coherence) length, assimilated to the average grain size of the tetragonal phase. To estimate the coherence length, we employed the integral breadth method [41]. The method calculates the volume-averaged coherence length G*_hkl_* from two parameters issued from the full-profile fitting: (a) the linewidth FWHM of superlattice L1_0_ Bragg peaks belonging to (hkl) = (001), (110), (111) and (200) planes and (b) the Bragg peak asymmetry, as revealed by the mixing parameter (or the proportion of Lorentz-type function in the overall pseudo-Voigt profile function) of each Bragg line. The coherence length was calculated then by averaging the G*_hkl_* values of all superlattice reflections for the samples annealed at temperatures from 450 °C up to 600 °C, i.e., for temperatures where the tetragonal phase is predominant, as seen in Figure 4. It was obtained that the coherence length increases from 63 nm for the sample annealed during the synchrotron XRD study at 450 °C to about 75 nm for the sample annealed during the synchrotron XRD study at 600 °C.

**Figure 6:**
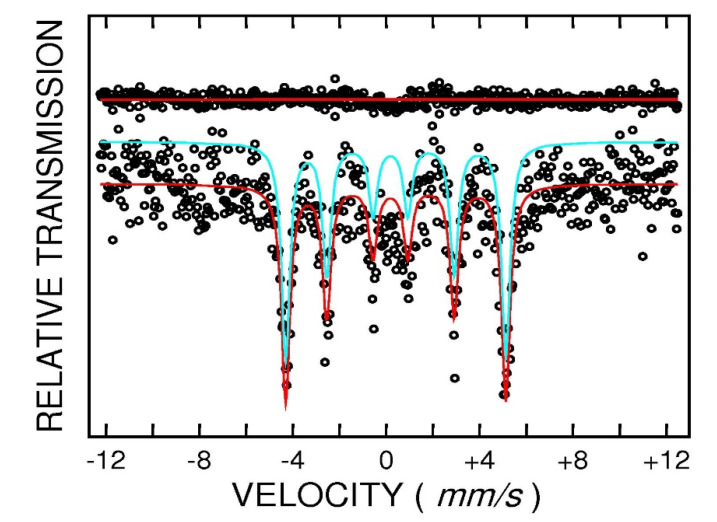
^57^Fe Mössbauer spectrum of the Fe_55_Pt_45_ sample annealed at 400 °C.

Additional information regarding structural features of the annealed FePt samples was furnished by means of Mössbauer spectroscopy. The Fe_55_Pt_45_powder sample annealed at 400 °C for 1 h was measured using ^57^Fe Mössbauer spectroscopy. The spectrum was recorded at 27 °C in transmission geometry in a velocity range of −12/+12 mm/s, and it is shown in Figure 6. The transmission pattern reveals the shape of a convoluted magnetic sextet, typical for ferromagnetic materials. Taking into account the large absorption factor for X-rays, exhibited by heavy metals such as Pt, and also the large Pt content in the sample, the magnitude of the measured absorption (Mössbauer effect) was surprisingly of a sufficient standard to accurately fit the spectrum. In agreement with the structural findings, we assumed that there are two unequivalent Fe ions in the chemical environment, one with cubic symmetry, attributable to the residual cubic FePt phase, and the other one with tetragonal symmetry, attributable to the ordered tetragonal FePt phase. Satisfactory fitting with two individual sextets provided us with the hyperfine parameters of the two sublattices: isomer shift (IS) = 0.29(2) mm/s (measured relative to metallic Fe), quadrupole splitting (QS) = 0.23(3) mm/s and hyperfine field (HF) = 27.8(1) T for the first component (I) and IS = 0.21(4) mm/s, QS = 0.01(2) mm/s and HF = 30.2(1) T for the second component (II). Taking into account the large value of the quadrupole splitting, which is typical for a tetragonally distorted cubic Fe environment, as well as the value of the hyperfine field, the first component is attributed to the L1_0_ phase. The near-zero quadrupole splitting of the second component is typical for a cubic Fe environment and, taking also into account the larger hyperfine field than in the first component, we can conclude that the second component is attributed to the fcc FePt phase. In what follows, we will show that the coexistence of cubic and tetragonal phases obtain more fundament from the magnetic measurements.

**Figure 7:**
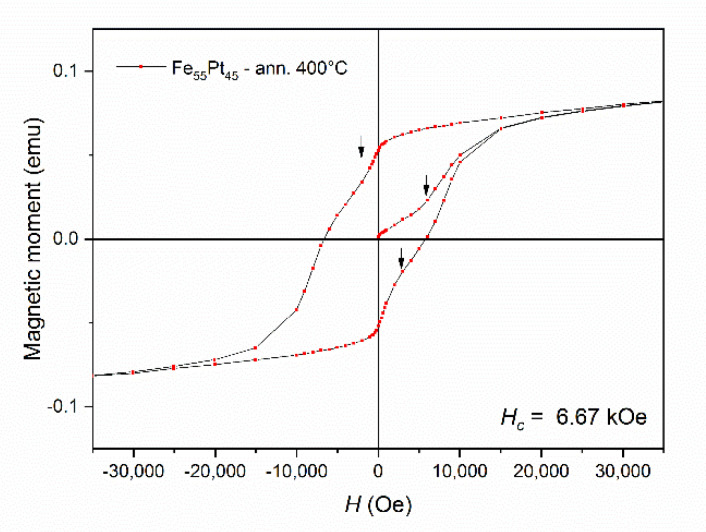
Initial magnetization and hysteresis loop of the sample Fe_55_Pt_45_ annealed at 400 °C.

The initial magnetization and hysteresis loop of the sample Fe_55_Pt_45_ annealed at 400 °C (Figure 7) were recorded using a Quantum Design SQUID magnetometer with the applied field parallel to the sample plane. The magnetic field was applied up to 50 kOe and measurements were taken at 27 °C. Initial magnetization shows quite slow approach to saturation, and an inflection point is observed on the magnetization curve at about 6 kOe. The curve continues its slow approach to saturation; however, this is not reached up to the maximum applied field of 50 kOe. The hysteresis loop shows considerably high remanent magnetization of about 65% of the maximum magnetization value and, more interestingly, shows a considerably high value of coercivity (*H_c_* = 6.67 kOe). Such values are consistent with the predominance of a hard magnetic phase in the sample and witness the formation of the L1_0_ phase at quite low temperatures of phase transformation (400 °C). It is to be noted that usually in FePt alloys, temperatures higher than 550 °C are needed in order to promote the formation of the L1_0_ phase, and this value is even higher in the case of FePt nanoparticles. To our knowledge, incipient formation of the L1_0_ phase at temperatures lower than 400 °C (around 300 °C) has only been reported by Wang and Barmak [42] in ultrathin FePt films co-sputtered on hot substrates. Two more inflection points, signaled by arrows on the graph, are observed on the hysteresis loop. These inflection points indicate the presence of a second magnetic phase, with significantly lower coercivity but high magnetization. This second magnetic phase is clearly softer than L1_0_ and all clues point to the fact that it is indeed the *fcc* FePt phase that was identified both in XRD and the Mössbauer spectrum. 

**Figure 8:**
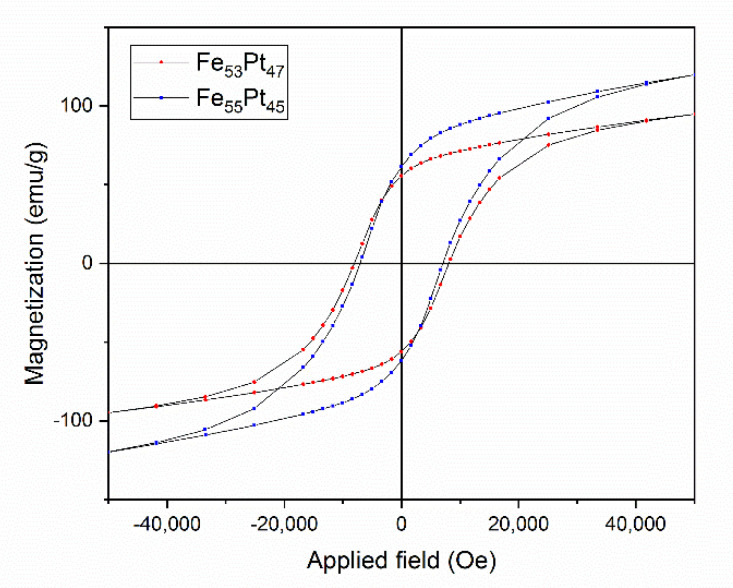
The 27 °C hysteresis loops of the Fe_55_Pt_45_ and Fe_53_Pt_47_ samples annealed at 500 °C.

While the coexistence of the two magnetic phases was confirmed by complementary investigations (XRD, MS, SQUID) in the Fe_55_Pt_45_ sample annealed at 400 °C, these two magnetic phases are not exchange coupled, as proven by the existence of the inflection points in the initial magnetization and the hysteresis loop. In contrast, the inflection points in the hysteresis loops allow in some cases determination of magnetic parameters of each individual magnetic sublattice. Their occurrence means that the different magnetic components—in our case the tetragonal (hard) and cubic (soft) magnetic phases—are not completely exchange coupled, and annealing at a higher temperature might be needed for achieving full exchange coupling of the two phases. For this purpose, we measured hysteresis loops of the Fe_55_Pt_45_ and Fe_53_Pt_47_ samples annealed at 500 °C. The two loops, recorded in same conditions as for the sample annealed at 400 °C (Quantum Design SQUID with a magnetic field of up to 50 kOe applied parallel to the sample plane at 27 °C), are shown in Figure 8. Contrary to the case of the sample annealed at 400 °C, here, the hysteresis loops of both samples show no inflection points and the allure of the hysteresis loop resembles what a single magnetic phase would look like. As in Figure 7, the curves exhibit a considerably slow approach to saturation with no inflection points. The saturation has not been reached for neither samples up to the maximum applied field of 50 kOe. The hysteresis loops of both Fe_55_Pt_45_ and Fe_53_Pt_47_ samples annealed at 500 °C show an even larger value of coercivity than the Fe_55_Pt_45_ sample annealed at 400 °C. While the values of coercive field are not very different between the two samples, there is a large difference in the maximum magnetization values (assimilated to *M_s_*) obtained at 50 kOe. The magnetic parameters recorded for the Fe_55_Pt_45_ sample annealed at 500 °C were *H_c_* = 7.05 kOe and *M_s_* = 119 emu/g. The same parameters recorded for Fe_53_Pt_47_ sample annealed at 500 °C were *H_c_* = 8.21 kOe and *M_s_* = 97 emu/g. The diminished magnetization at 50 kOe for the Fe_53_Pt_47_ sample annealed at 500°C can be explained by the smaller amount of the ordered tetragonal L1_0_ phase, which was already formed at 400 °C (see Table 1). After annealing at 400 °C, there was a 6% difference between the abundances of the already-formed L1_0_ phase in the two samples; thus, it is quite probable that this trend was preserved also after annealing at 500 °C. Between the two alloys with a slightly different composition, a delay in the incipiency of the tetragonal phase for the sample with the lower Fe content was observed. Synchrotron XRD studies proved that the amount of the L1_0_ phase formed in Fe_53_Pt_47_ sample at 500 °C annealing is about 5% lower than the amount of L1_0_ already formed in the same annealing conditions for the Fe_55_Pt_45_ sample. 

In order to quantify the potential magnetic performance of these alloys, we also calculated the maximum energy product (BH)_max_, a parameter which measures the amount of magnetic energy usable in these magnets in various applications. Being essentially proportional to the area encompassed by the hysteresis loops, (BH)_max_ was calculated from the experimental magnetization vs. applied field values and was found to be 19.8 MGOe for the Fe_55_Pt_45_ sample annealed at 500 °C and 18.4 MGOe for the Fe_53_Pt_47_ sample annealed at 500 °C. These values are situated towards the higher limits reached in the literature for FePt films—for instance, Shen et al. in [43] reported values of about 20 MGOe for 300 nm thick FePt thin films annealed at 500 °C.

These findings lead to the creation of a potentially exchange-coupled nanocomposite magnet where both soft and hard magnetic phases coexist and yield a large enough maximum energy product that is comparable with existing permanent magnets.

## 4. Conclusions

Two alloys of a composition slightly off-stoichiometric from the equiatomic FePt system were synthesized by ball milling in a dynamic, rotation-switched regime of a planetary ball-milling device from high purity elemental powders dispersed in liquid carrier (hexane) in order to avoid Pt adherence to the balls. While the as-milled state of samples Fe_53_Pt_47_ and Fe_55_Pt_45_ show a face-centered cubic A1 crystalline symmetry, the annealing at 400 °C produced multiple additional Bragg lines in the X-ray diffractograms. The occurrence of additional so-called superlattice peaks as well as the splitting observed for some of the Bragg peaks of the initial cubic symmetry due to tetragonal distortion showed that the L1_0_ phase FePt is formed after annealing at 400 °C, a temperature lower than what is usually needed to form the L10 phase in conventional FePt alloys and nanoparticles. Quantitative full-profile analysis of the XRD diffractograms allowed us to prove a high amount of the L1_0_ phase (72% and 78%, respectively, for Fe_53_Pt_47_ and Fe_55_Pt_45_), while ^57^Fe Mössbauer spectroscopy brought further proof for the two-phase behavior. Two magnetic sublattices were identified from the fitting of the Mössbauer spectrum with significantly different quadrupole splitting and hyperfine field values. Magnetometry measurements provided further evidence of the coexistence of the hard magnetic tetragonal L1_0_ and soft magnetic cubic FePt phases, with strong predominance of the hard magnetic phase. This was proven by the existence of the inflection points in the hysteresis loops, which is a sign of the interplay between two magnetic phases, one hard magnetic and another one soft magnetic. For the Fe_53_Pt_47_ and Fe_55_Pt_45_ samples annealed at 500 °C, the two magnetic phases became fully exchange coupled as witnessed by the lack of inflection points in the hysteresis loops. As a consequence, large coercivity (up to 8.2 kOe) and significant remanent magnetization were obtained, leading to a maximum energy product of up to 19.8 MGOe, which is comparable with those of current permanent magnets. These results provide an interesting method to obtain an exchange spring nanocomposite magnet, which is promising in view of use of such materials as a future class of rare earth-free permanent magnets.

## Figures and Tables

**Table 1:** Lattice parameters and average grain sizes obtained from full-profile MAUD analysis.

Sample	Phase	Lattice Parameters (Å)	Average Grain Size (nm)	Amount (%)
Fe_53_Pt_47_—as-milled	*fcc* FePt	*a* = 3.8037 ± 0.0002	*D*=22 ± 3	100
Fe_53_Pt_47_—anneal. 400 °C	*fcc* FePt	*a* = 3.7972 ± 0.0002	*D*=25 ± 4	27.2
	L1_0_ FePt	*a* = 3.8383 ± 0.0001	*D*=38 ± 7	72.8
		*c* = 3.7228 ± 0.0016		
Fe_55_Pt_45_—as-milled	*fcc* FePt	*a* = 3.8045 ± 0.0004	*D*=27 ± 2	100
Fe_55_Pt_45_—anneal. 400 °C	*fcc* FePt	*a* = 3.7925 ± 0.0003	*D*=32 ± 3	21.3
	L1_0_ FePt	*a* = 3.8249 ± 0.0004	*D*=49 ± 5	78.7
		*c* = 3.7116 ± 0.0013		
